# The Affordable Care Act: policy predictors of integrated care between Hispanic-serving and mainstream mental health organizations

**DOI:** 10.1186/s12913-021-06198-6

**Published:** 2021-02-28

**Authors:** Robert Rosales, Rocío Calvo

**Affiliations:** 1 Department of Behavioral & Social Sciences, Center for Alcohol and Addictions Studies, Brown University School of Public Health, 121 South Main Street, 4th Floor, Providence, RI 02903 USA; 2 Boston College, School of Social Work, McGuinn Hall, 140 Commonwealth Avenue, Chestnut Hill, MA 02467 USA

**Keywords:** Hispanics, Integrated care, Affordable care act, Community health centers, Hispanic-serving organizations

## Abstract

**Background:**

The Patient Protection and Affordable Care Act increased funding for integrated care to improve access to quality health care among underserved populations. There is evidence that integrated care decreases inequities in access and quality of mental health care among Hispanic clients. Increasing integrated care at Hispanic-Serving Organizations may help to eliminate mental health service disparities among Hispanic clients.

**Method:**

Using organizational responses from the 2014 and 2016 waves of the National Mental Health Service survey, this study conducted multivariate logistic analyses to assess whether the ACA policies related to integrated care increased the provision of integrated addictions treatment and primary care at mental health Hispanic-Serving Organizations, relative to Mainstream Organizations.

**Results:**

Findings showed that Hispanic-Serving Organizations (54.4%) were less likely to provide integrated health services than Mainstream Organizations (59.1%) after the ACA. However, federal funding to help organizations transition into integrated care services (AOR = 1.74, *p* = 0.01) and accepting Medicaid payments (AOR = 1.59, *p* = 0.01) increased the provision of integrated care services at Hispanic-Serving Organizations over time.

**Conclusions:**

Health care policies that increase funding to adopt integrated health services at community Hispanic-Serving Organizations may help decrease inequities in mental health access for Hispanics in the United States.

## Background

Research shows that there are persistent disparities in health care services between Hispanic-Serving Organizations (HSOs) and Mainstream Organizations (MOs) [1, 2]. HSOs, defined as organizations with more than 20% of Hispanic clients [3], tend to score low in accessibility and quality of health care [4]. Integrated care, a collaborative model of health care delivery, has been shown to increase healthcare quality among Hispanic clients [5]. However, less than one-third of HSOs provide integrated health care [3]. Increasing the provision of integrated care at HSOs may ameliorate disparities in access to quality health care among Hispanics [5].

The Patient Protection and Affordable Care Act (ACA) promoted the adoption of integrated care to increase the quality of health care that Americans receive. To that end, the ACA fostered the integration of mental health care into primary care and into addictions treatment, and increased funding for health care organizations that wished to adopt integrated services [6]. Little is known about the effect of these measures on Hispanic-Serving Organizations (HSOs).

### Integrated health care and mental health care among Hispanic clients

Integrating addictions treatment and primary care into mental health care is a practical approach to increasing access to quality mental health care among Hispanics, especially for those with serious mental illness [5, 7–9]. Integrated care is a collaborative model of health care delivery that involves multiple levels of collaboration. Rather than merely placing physical and behavioral health clinicians in the same setting, integrated care encourages multidisciplinary teams’ cooperation in the creation of assessments, interventions, and the delivery of services. Therefore, integrating mental health services into primary care settings and into addiction services helps address barriers associated with referrals, transportation, cost, and the difficulties associated with scheduling various services at multiple locations [10].

This collaborative way of providing health care has shown promise in decreasing disparities in access to mental health care among Hispanic clients by normalizing the use of behavioral health [11]. Bridges and colleagues [5] found that Hispanic clients who received behavioral services in integrated settings experienced reduced mental health symptoms at a similar rate as non-Hispanic whites. Participants also reported high levels of satisfaction with the quality of care experienced in integrated care settings. These findings suggest that integrated care may reduce inequities in access to quality mental health care for Hispanic clients.

### Integrated health care and the Affordable Care Act (ACA)

The ACA intended to increase access to quality health care, including mental health care, among underserved populations. To this end, the ACA: (1) provided funding to community health centers to facilitate the adoption of integrated care, and (2) increased access to health care by expanding Medicaid eligibility.

To facilitate the adoption of integrated care, the ACA created two major grants. The first grant assigned $105.8 million to the Health Resources and Services Administration (HRSA) to assist community health care centers in providing integrated health care services to populations with severe physical and mental illnesses [12]. The second grant established the $11 billion Community Health Centers Fund (CHCF), which helped increase community health centers’ capacity to provide integrated care [13]. This CHCF's primary purpose was to assist community health centers with the infrastructure, processes, and staff necessary to transition into integrated health care services [14].

The expansion of the criteria to qualify for Medicaid was the chief strategy to increase health coverage among populations that lacked health insurance, including Hispanics. However, some states challenged the mandatory expansion of Medicaid, which led the Supreme Court to declare it optional [15]. By 2014, the year in which the ACA had to be fully implemented, only 25 states had adopted the expansion of Medicaid [16]. The discrepancy in available Medicaid funds between expansion and non-expansion states created a fractured system, where organizations in expansion states benefited from a larger pool of Medicaid payments than organizations in non-expansion states. As a result, health care organizations in expansion states saw a decrease in uncompensated care stemming from an increase in Medicaid coverage of low-income patients [17–20]. Extra funds incentivized mental health care organizations in expansion states to invest in the transition to integrated care. Additionally, mental health care organizations could use new bundled Medicaid payments for integrated care. For instance, organizations could adopt Medicaid bundled payments for a package of services that included both behavioral and physical health care [21, 22]. Health care organizations took advantage of the ACA’s new bundled payments option, which allowed them to be reimbursed for a package of services rather than charge services separately.

### Aim of the study

The ACA promoted mental health care integration into primary care and into addictions treatment to increase access to quality health care. It is unknown how these provisions impacted HSOs. The current study investigates: 1) Whether the provision of integrated primary care and addictions treatment increased in HSOs between 2014 and 2016, and 2) The impact of the ACA measures for the promotion of integrated care in HSOs.

## Methods

### Study Design & Survey Tool

We conducted multivariate logistic regression analyses with secondary cross-sectional data from the 2014 and 2016 National Mental Health Services Survey (NMHSS). The N-MHSS is a publicly available national census of mental health organizations in the US. The N-MHSS, which has been conducted annually since 2014, uses paper, web-based, and telephone-assisted questionnaires to gather organizations’ clients, structure, and service characteristics. The N-MHSS does not collect data from military treatment facilities, jails or prisons, residential treatment facilities without specialty behavioral services, and individual and small group practices not licensed as mental health clinics [23, 24].

### Sample

We selected the 2014 and 2016 waves of the N-MHSS because they are the only publicly available data waves that included the ethnoracial characteristics of the population served at the mental health organizations. The Center for Behavioral Health Statistics and Quality (CBHSQ) of the Substance Abuse and Mental Health Services Administration (SAMHSA), and the US Department of Health and Human Services requested all their private and public mental health organizations throughout the 50 US states to take part in the N-MHSS. Each organization’s directors completed the 2014 N-MHSS survey between March 2014 and January 2015 and the 2016 N-MHSS between March 2016 and January 2017.

In terms of inclusion and response rate, 88.1% of the 16,687 organizations identified in 2014 completed the survey. Over 10% of the organization were excluded afterward because they only provided administrative services, were included in other organization counts, or were determined to be out-of-scope by the survey administrators. Of the 13,983 organizations identified in 2016, 91.1% completed the survey, and 4.1% were excluded afterward because they only provided administrative services or were included in other organization counts. The current study excluded 5732 organizations because they did not report their Hispanic population, which resulted in a final analytic sample of 19,314 organizations. The analytical sample included 9857 organizations from the 2014 N-MHSS and 9457 from the 2016 N-MHSS.

Unique case identifiers for each of the organizations were not publicly available. Therefore, we used a repeated cross-sectional design to merge both waves into one dataset, which is an established method to combine multiple waves of data into a single dataset when there are no unique identifiers [25].

### Dependent variables

#### Integrated health care

We used two variables to assess whether the mental health organizations provided integrated addictions treatment and integrated primary care. The first variable derived from a question that captured whether organizations provided any of 14 mental health treatments using the following question, “Which of these mental health treatment modalities are offered at this facility, at this location?” Organizations that reported providing integrated substance use treatments were coded as (1), while organizations that did not report this service were coded as (0). The second variable was derived from a question that captured whether organizations provided any of 26 services using the following question, “Which of these services and practices are offered at this facility, at this location?” Organizations that reported providing integrated primary care services were coded as (1), while organizations that did not report this service were coded as (0).

### Independent variables

#### ACA measures

##### Federal Grants

Organizations were asked to respond to the following question: “Which of the following types of client payments, insurance, or funding are accepted by this facility for mental health treatment services?” The federal grants variable used responses on this question to capture whether organizations (1) received, or (0) did not receive these community mental health block grants. Community mental health block grants were defined as funding from a Health Resources and Services Administration (HRSA) grant or Community Health Centers Fund (CHCF) grant.

##### Organization in an expansion state

Using Medicaid expansion state data from the Kaiser Family Foundation [16], a variable was created that measured whether organizations were located in Medicaid (1) expansion or (0) non-expansion states.

##### Organizations take Medicaid payments

This variable, taken from the N-MHSS survey, captured whether the organizations accepted Medicaid payments. Responses were coded as (1) Yes, or (0) No.

### Control variables

Each model included a set of client and organizational characteristics. The ownership variable categorized organizations into (0) public, (1) private non-profit, and (2) private for-profit. We also adjusted for whether organizations were community mental health centers (1) Yes, or (0) No; and for their outpatient size, which captured the total number of clients served at the organization in April of the year the data was collected. Outpatient size ranged from 0 (none) to 12 (more than 1500). In terms of the characteristics of the clients served at each organization annually, we controlled for the percentage of children (individuals 17 years or younger), adults (individuals between 18 to 64 years of age), and older adults (people 65 years and older), as well as for the percentage of female clients. Client characteristic variable responses ranged from 0 (none) to 7 (More than 75%).

### Data analysis

We conducted a three-step data analysis using Stata, Statistical Software 15.1. First, we used prior literature on ethnoracial minority-serving organizations to categorize organizations into HSOs (i.e., more than 20% Hispanic clients) and MOs (i.e., 20% or fewer Hispanic clients) [3]. We included MOs in the models because comparing the findings for HSOs and MOs allows for a better understanding of the ACA’s impact on providing integrated addictions treatment and primary care at HSOs. On a second step, we conducted a series of bivariate analyses to assess potential differences between HSOs and MOs concerning the study’s variables. The third step consisted of two sets of multivariate logistic analyses. The first set of regressions identified the predictors associated with providing integrated addictions treatment at mental health HSOs and MOs. The second set of regressions identified the predictors related to providing integrated primary care at mental health HSOs and MOs. Each set of regressions was conducted separately for HSOs and MOs. We calculated adjusted odds ratios (AOR) by controlling for the remaining predictor variables (Medicaid expansion, Medicaid payments accepted, community mental health block grant, and year), and for control variables (ownership, community mental health center, organization size, % youth, % adults, % older adults, and % female served annually).

#### Interaction terms

Interactions assessed whether the ACA’s impact in the provision of integrated care 1) was different in expansion versus non-expansion states, and 2) changed from 2014 to 2016. Each interaction model was run separately and adjusted by controlling for the main effects of the predictor variables (Medicaid expansion, Medicaid payments accepted, community mental health block grant, year) and of the control variables (ownership, community mental health center, organization size, % youth, % adults, % older adults, and % female served annually).

## Results

### Characteristics of the sample

Preliminary findings from this study were reported elsewhere [26]. Table 1 reports the sample characteristics for the HSOs and MOs and the bivariate analysis results. MOs were significantly more likely to provide integrated addictions services than HSOs, χ^2^(1, *N* = 19,244) = 21.65, *p* < 0.001. Specifically, 59.1% of MOs provided integrated addictions services relative to 54.4% of HSOs. No significant differences emerged between HSOs and MOs concerning the provision of integrated primary care.

**Table 1:** Sample characteristics stratified by Hispanic-serving (HSO) and Mainstream organizations (MO) (*N* = 19,314)

Variables	HSO (*N* = 2803)		MO (*N* = 16,511)		*p*-values
	n	%	n	%	
*Integrated Care Variables*					
Integrated addictions treatment					< 0.001
Yes	1520	54.4	9725	59.1	
No	1273	45.6	6726	40.9	
Integrated primary care					0.549
Yes	619	22.2	3734	22.7	
No	2166	77.8	12,686	77.3	
*ACA Provisions*					
Medicaid expansion					< 0.001
Expansion state	2174	77.6	9418	57.0	
Non-expansion state	629	22.4	7093	43.0	
Medicaid payments accepted					0.978
Yes	2501	91.0	14,706	90.9	
No	249	9.0	1467	9.1	
Community mental health block grants					< 0.001
Yes	845	38.2	5630	42.9	
No	1365	61.8	7505	57.1	
*Control Variables*					
Community mental health center					< 0.001
Yes	703	25.1	4923	29.8	
No	2100	74.9	11,588	70.2	
Ownership					< 0.001
Public/other	537	19.2	3312	20.1	
Private non-profit	1970	70.3	10,398	63.0	
Private for-profit	296	10.5	2801	16.9	
	HSO		MO		
	M [Category^a^] (SD)		M [Category^a^] (SD)		*p*-values
Organization size	7.7 [101 to 250] (2.8)		7.4 [76–100] (2.9)		< 0.001
% Youth clients	3.6 [31 to 40%] (2.8)		2.8 [21 to 30%] (2.5)		< 0.001
% Adult clients	4.5 [41 to 50%] (2.6)		5.3 [41 to 50%] (2.2)		< 0.001
% Older adult clients	0.9 [1 to 10%] (1.1)		1.2 [1 to 10%] (1.3)		< 0.001
% Female clients	5.1 [41 to 50%] (1.2)		5.2 [41 to 50%] (1.3)		< 0.001

*Notes.* Percentages are presented for nominal variables and means and standard deviations for ordinal/continuous variables. All variables were included in 2014 and 2016 N-MHSS. Mental-health Hispanic-serving organizations = more than 20% of Hispanic clients; Mainstream organizations = 20% or fewer of Hispanic clients

^a^ Categories were determined by rounding the decimal to the nearest whole number

Turning to the association between the ACA measures and the provision of integrated care, MOs were more likely (42.9%) than HSOs (38.2%) to receive federal block grants for the adoption of integrated care, χ^2^(1, *N* = 15,345) = 16.61, *p* < 0.001. By contrast, almost 78% of HSOs were located in expansion states relative to 57% of MOs, χ^2^(1, *N* = 19,314) = 420.43, *p* < 0.001. No significant differences emerged between HSOs and MOs concerning the acceptance of Medicaid payments, χ^2^(1, *N* = 18,923) > 0.01, *p* = 0.978.

Concerning the control variables, more MOs (29.8%) than HSOs (25.1%) self-identified as community health centers, χ^2^(1, *N* = 19,314) = 26.04, *p* < 0.001. HSOs were larger and served more youth than MOs, concentrating on adult, older adult, and female clients. In terms of ownership, HSOs were more likely to be non-profit organizations, while MOs were more likely to be for-profit organizations.

#### Integrated addictions services in mental health care organizations

Table 2 presents the logistic regression analyses findings concerning the association between the ACA measures and the provision of integrated addiction services. We observed a significant decrease in the provision of integrated addictions care both at HSOs (AOR = 0.69, *p* < 0.001) and MOs (AOR = 0.77, *p* < 0.001) from 2014, the year the ACA was implemented, to 2016. HSOs and MOs had 31 and 23% lower odds, respectively, of providing integrated addictions treatment 2 years after implementing the ACA.

**Table 2:** Logistic regression analysis on integrated addictions treatment stratified by HSOs and MOs (2014 to 2016)

	Hispanic-serving Organizations (*N* = 2159)			Mainstream Organizations (*N* = 12,502)		
	AOR	95% CI	*p*-value	AOR	95% CI	*p*-value
*Year*						
2016	0.69	0.58–0.83	< 0.001	0.77	0.72–0.83	< 0.001
Medicaid expansion	0.99	0.78–1.24	0.898	0.93	0.86–1.00	0.062
Medicaid payments accepted	1.59	1.15–2.19	0.005	1.01	0.89–1.15	0.859
Community mental health block grant	1.60	1.31–1.94	< 0.001	1.75	1.61–1.90	< 0.001
*Medicaid Expansion Interaction Terms*						
Medicaid expansion x Medicaid payments accepted	1.09	0.50–2.37	0.831	1.16	0.91–1.47	0.242
Medicaid expansion x CMHC	1.45	0.90–2.32	0.129	0.87	0.74–1.02	0.080
Medicaid expansion x CMHBG	1.25	0.80–1.95	0.321	1.01	0.86–1.17	0.939
*Time Interactions*						
Medicaid expansion × 2016	0.68	0.44–1.05	0.077	0.85	0.73–0.98	0.028
Medicaid payments accepted × 2016	0.68	0.36–1.27	0.224	0.84	0.66–1.06	0.141
CMHC × 2016	1.74	1.14–2.66	0.010	0.89	0.76–1.05	0.159
CMHBG × 2016	1.38	0.95–2.02	0.095	0.90	0.78–1.05	0.177

Note. *AOR* Adjusted Odds Ratio. All models accounted for control (ownership, organization size, community mental health center, % youth, % adults, % older adults, and % female), Medicaid expansion (Medicaid expansion, Medicaid payments accepted, Community mental health block grant), and time variables. Interaction models controlled for all main effects, including control variables. Interaction terms were conducted separately from each other. Year: 2010 is the reference category. Ownership: public departments is the reference categories. *CMHC* Community mental health center, *CMHBG* Community mental health block grant

In terms of the association between the ACA measures and integrated addictions services, results showed that HSOs (AOR = 1.60, *p* < 0.001) and MOs (AOR = 1.75, *p* < 0.001) that received federal block grants were more likely to transition into integrated care. Whether organizations were located or not in expansion states was not significantly associated with integrated care provision. By contrast, accepting Medicaid payments increased the chances of providing integrated addictions services at HSOs (AOR = 1.59, *p* = 0.005), but not at MOs (AOR = 1.01, *p* = 0.859).

Concerning interaction effects, after controlling for main effects and control variables, we observed significant differences only on the impact of the ACA measures over time. There was a significant decrease in the provision of integrated services in MOs located in expansion states (AOR = 0.85, *p* = 0.002). MOs in expansion states had 15% lower odds in 2016 than in 2014 of providing integrated addictions services. By contrast, community mental health HSOs had 74% greater odds of providing integrated addictions services in 2016 than in 2014 (AOR = 1.74, *p* = 0.010).

#### Integrated primary care in mental health care organizations

Table 3 presents the findings for the logistic regression analysis for the provision of integrated primary care services. Results showed no significant change from 2014 to 2016 in the provision of integrated primary care services at HSOs (AOR = 1.23, *p* = 0.068) or at MOs (AOR = 0.98, *p* = .674).

**Table 3:** Logistic regression analysis on integrated primary care stratified by HSOs and MOs (2014 to 2016)

	Hispanic-serving organizations (*N* = 2173)			Mainstream organizations (*N* = 12,944)		
	AOR	95% CI	*p*-value	AOR	95% CI	*p*-value
*Year*						
2016	1.23	0.99–1.53	0.068	0.98	0.90–1.07	0.674
Medicaid expansion	0.89	0.68–1.16	0.371	1.00	0.91–1.10	0.983
Medicaid payments accepted	1.10	0.72–1.69	0.664	0.59	0.51–0.69	< 0.001
Community mental health block grant	1.97	1.57–2.49	< 0.001	1.26	1.14–1.39	< 0.001
*Medicaid Expansion Interaction Terms*						
Medicaid expansion x Medicaid payments accepted	3.16	1.26–7.93	0.014	0.90	0.69–1.17	0.437
Medicaid expansion x CMHC	1.42	0.83–2.43	0.199	0.95	0.79–1.15	0.610
Medicaid expansion x CMHBG	1.08	0.65–1.80	0.775	1.24	1.04–1.49	0.017
*Time Interactions*						
Medicaid Expansion × 2016	0.62	0.37–1.02	0.061	0.99	0.82–1.18	0.868
Medicaid payments accepted × 2016	1.15	0.50–2.62	0.742	0.97	0.74–1.26	0.804
CMHC × 2016	1.43	0.89–2.30	0.145	1.10	0.91–1.33	0.312
CMHBG × 2016	1.79	1.15–2.79	0.010	0.97	0.81–1.16	0.729

Note. *AOR* Adjusted Odds Ratio. All models accounted for control (ownership, organization size, community mental health center, % youth, % adults, % older adults, and % female), Medicaid expansion (Medicaid expansion, Medicaid payments accepted, Community mental health block grant), and time variables. Interaction models controlled for all main effects, including control variables. Interaction terms were conducted separately from each other. Year: 2010 is the reference category. Ownership: public departments is the reference categories. *CMHC* Community mental health center, *CMHBG* Community mental health block grant

Turning to the impact of the ACA measures on the provision of integrated primary care services, HSOs (AOR = 1.97, *p* < 0.001) and MOs (AOR = 1.26, *p* < 0.001) that received federal funding in the form of community mental health block grants were more likely to provide integrated primary health care services. Additionally, MOs that accepted Medicaid payments were significantly less likely to offer integrated primary care services (AOR = 0.59, *p* < 0.001).

After controlling for main effects and control variables, the interaction terms between Medicaid expansion and the ACA measures showed that HSOs in expansion states that accepted Medicaid payments were more likely to provide integrated primary care services (AOR = 3.16, *p* = 0.014). Turning to the interaction on the impact of the ACA over time, after adjusting for main effects and control variables, HSOs that received community mental health block grants had greater odds of providing integrated primary care 2 years after the implementation of the ACA (AOR = 1.79, *p* = 0.010).

## Discussion

To increase the quality of health care in the US, the Patient Protection and Affordable Care Act (ACA) promoted the adoption of integrated care. To that end, the ACA fostered the integration of mental health care into primary care and addictions treatment, and increased funding for health care organizations that wished to adopt integrated services. This study investigated: 1) whether integrated care after the ACA increased in HSOs relative to MOs 2) the impact of the ACA measures for the promotion of integrated care in HSOs relative to MOs.

### Trends in integrated care at HSOs and MOs after the ACA

Results showed a significant decrease in the provision of integrated addictions treatment in HSOs and MOs 2 years after implementing the ACA. No significant differences were observed in the provision of integrated primary care between organizations. The decrease in the provision of integrated addictions treatment may be in response to challenges in implementing the reform. Health care organizations reported problems stemming from inadequate funding, technology gaps, and shortages of trained integration clinicians [27]. For instance, there is evidence that the greater cost of providing care to individuals with serious comorbid illnesses prevented organizations from adopting and keeping integrated models of care after the ACA [13]. Integrated health care settings require costly new systems to handle the provision of care, as well as systemic changes in organizational structures to process insurance claims and payments [28]. These changes forced some organizations to assume the burden of costly staff training, making the provision of integrated care unsustainable [21, 22].

### Differences in the trends in integrated care between HSOs and MOs

While there was a decrease in the provision of integrated addictions treatment at MOs and HSOs after the ACA, the decline was substantially larger for HSOs. This is consistent with prior research on integrated care disparities between HSOs and MSOs [1, 2]. Specifically, less than a third of HSOs nationwide provide integrated mental health and addictions treatment services, relative to almost half of MOs [3]. Providing integrated substance use care increases the demand for professionals who can provide these services. Yet, there is a shortage of clinicians trained to provide integrated addictions services [29], especially among Spanish-speaking clients who have enormous difficulties finding ethnic-concordant providers who can provide services in Spanish [30]. Yet, there is evidence that substance misuse deaths have increased since the ACA’s adoption [31–34]. Marijuana use rates among Hispanic youth, one of the central populations served at HSOs, have significantly increased in the last few years [35, 36]. Although integrated care may decrease disparities concerning access to quality mental health care among Hispanic clients [5], they are significantly less likely to live near organizations that provide integrated care [37]. Barriers to integrated care is a critical issue among Hispanics with severe mental illness who have historically faced limitations with overall access to care.

### ACA provisions to increase access to integrated care

Accepting Medicaid payments and receiving block grants were associated with increased provision of integrated addictions treatment at HSOs. These findings align with prior evidence showing that organizations accepting Medicaid payments have financially benefitted from the increase in insured clients [19]. Medicaid payments account for more than 25% of mental health services in the US. Meanwhile, the bundled payments adopted with the ACA provided new opportunities for the integration of addictions treatment in mental health care organizations [38]. With the reimbursement from these new payment methods, HSOs can provide evidence-based integrated care, including transition care [38]. Before the ACA, several obstacles made it difficult for mental health care organizations to charge for integrated services. Health insurance providers, including State Medicaid Plans, did not allow health care organizations to bill for more than one type of services (e.g., behavioral and primary care) within the same day [39, 40]. This forced organizations not to bill Medicaid, turning instead to private insurers to cover these integrated services [41].

Our interaction terms showed that over time HSO community mental health centers were more likely to adopt integrated addictions services and integrated primary care. Similarly, HSOs that accepted Medicaid payments and that were in Medicaid expansion states increased integrated primary care provision. This suggests that the ACA’s strategy of facilitating the payment of integrated services through increased federal funding for HSOs community health centers that wished to adopt an integrated care infrastructure was effective. Community mental health block grants have increased since the implementation of the ACA [42]. Our findings suggest that this funding helps close the gap on the provision of integrated care at HSOs.

## Limitations

Notwithstanding the strengths of this study, some limitations should be considered. First, since the N-MHSS did not include unique case identifiers, we could not identify the number of organizations that overlapped from the 2014 to 2016 N-MHSS. Since all organizations in this study came from the same database, there may be overlap between organizations in 2014 and 2016. Second, the use of a repeated cross-sectional design precludes us from drawing causal conclusions of the findings. Third, health care integration is a complex system. It is a healthcare delivery method where all practitioners must learn to work within the new integrated system [43]. Our use of two general questions about integrating addictions treatment and primary care may not capture the integration level involved in healthcare settings [44]. Finally, the term HSOs does not account for other essential services that would increase access to care among Hispanic clients, such as language concordance. However, the advantage of using this term is that it allows the assessment of how the ACA increased the provision of integrated care at mental health care organizations with larger proportions of Hispanic clients, even if they do not provide culturally congruent care.

## Conclusion

This study found that while HSOs provided fewer integrated behavioral health services 2 years after implementing the ACA, the federal funding allocated to help community health care centers transition into integrated care services and accepting Medicaid payments increased the provision of integrated care services at HSOs. Health care policies that help to increase funding for the adoption of integrated health services at community HSOs may help decrease inequities in access and quality of health care among Hispanics. This study helps decrease the shortage of research on how the ACA is associated with health care disparities at HSOs [45].

## Data Availability

The datasets generated during and/or analyzed during the current study are available in the Drug & Alcohol Services Information System repository, [https://wwwdasis.samhsa.gov/dasis2/nmhss.htm].

## Abbreviations

ACA: The Patient Protection and Affordable Care Act
HSOs: Hispanic-Serving Organizations
MOs: Mainstream Organizations
PCPs: Primary care physicians
N-MHSS: National Mental Health Services Survey
